# DEVELOPING EVIDENCE-BASED INSTRUMENTS TO TALK ABOUT LONELINESS

**DOI:** 10.1093/geroni/igz038.3047

**Published:** 2019-11-08

**Authors:** Eric Schoenmakers

**Affiliations:** Fontys University of Applied Sciences, Eindhoven, Netherlands

## Abstract

People, professionals and non-professionals, lonely and non-lonely, experience difficulties in talking about loneliness. Yet, talking about loneliness is important. Without conversation about loneliness, it is impossible to identify lonely persons and to assist them in a tailor-made way. The aim of this research project is to create evidence-based instruments to make talking about loneliness easier. From 2016 to 2019 (ongoing) six researchers held about 60-70 interviews with lonely persons, discussing feelings of loneliness and coping behavior. After the first 23 interviews with lonely older adults, a qualitative analysis on how to discuss loneliness was performed, resulting in a format for talking about loneliness and a topic list to help in conversations. These instruments were tested in the remaining interviews with lonely older adults and students, after which the format and topic list were updated. The format for talking about loneliness discusses guidelines for the inter human relationship, how to bring up the topic of loneliness, and what to discuss, e.g. feelings, timing, coping, consequences and taboo. ‘What to discuss’ is also addressed in the topic list. It is important to not be eager to help. Often, lonely people have felt so for a long time and considered many coping options. This illustrates complexity. Trying to ‘solve’ loneliness with oversimplified suggestions makes people feel that their situation is not taken seriously. These instruments emphasize the importance of true listening. The instruments can be used to train professionals and volunteers who want to discuss loneliness with lonely people.

